# Measuring relative telomere length: Is tissue an issue?

**DOI:** 10.18632/aging.100236

**Published:** 2010-11-23

**Authors:** Monica M. Gramatges, Alison A. Bertuch

**Affiliations:** Department of Pediatrics, Baylor College of Medicine, BCM320, Houston, TX 77030, USA

Telomeres, the specialized structures at chromosome ends, consist of long stretches of protein-bound TTAGGG repeats [1]. In certain cell types, such as germ cells and stem cell populations, the length of telomeric DNA is maintained by the enzyme telo-merase. Most somatic cells, however, lack sufficient telomerase; consequently, telomeric DNA shortens progressively with each cell division. Upon reaching a critically short length, proliferation is halted and cells often enter a state of senescence. Thus, progressive telomere shortening is thought to serve as a molecular clock for cellular replicative aging. Based upon this fundamental tenet of telomere biology, an ever-increasing number of studies spanning a wide spectrum of human conditions have utilized telomere length, typically measured in buccal cells or blood mono-nuclear cells, as a biomarker for cellular replicative age. Individuals with relatively short age-adjusted telomere lengths, due to a combination of inherent factors and environmental stressors, are considered to have acceler-ated cellular replicative aging, potentially resulting in increased disease susceptibility [2].

Telomere length is also used as a diagnostic tool in diseases characterized by fundamental derangements of telomere biology, such as dyskeratosis congenita (DC). DC is a rare genetic disorder stemming from a defect in telomere maintenance. This defect results in a broad and highly variable clinical phenotype consisting of predisposition to bone marrow failure and malignancy, a triad of mucocutaneous features, and a number of less frequent manifestations such as pulmonary fibrosis and liver disease [3]. In nearly all reported cases, affectted individuals have severely shortened telomeres, which can be directly attributed to mutations in genes encoding components of telomerase or a telomere-associated protein in approximately half of cases.

Hence, DC is considered the prototype of a heritable disorder of telomere maintenance. In DC, very short telomeres are observed across different classes of peripheral white blood cells, affecting both lymphoid subpopulations and granulocytes [4]. In contrast, in other inherited bone marrow failure syndromes (IBMFS), telomere length shortening is less pronounced and the effect is largely restricted to granulocytes. Rather than being due to an inherent defect in telomere maintenance, the short telomeres in granulocytes in these cases are thought to reflect accelerated progenitor/stem cell turnover secondary to bone marrow stress.

The utility of telomere length both as a biomarker for accelerated cellular replicative aging and as a diag-nostic marker for a constitutional defect in telomere maintenance solicits a question as to how telomere length varies in different tissues in conditions associated with accelerated cellular aging as compared to disorders where telomere length maintenance is compromised. In this issue of *Aging*, Gadalla, *et al*, begin to address this question by simultaneously examining relative telomere length in granulocytes, fibroblasts and buccal cells within individuals affected by DC and other IBMFS [5]. Although the number of subjects was small, strong correlations between blood, buccal cells, and fibroblasts were observed in the study population as a whole. When taken individually, however, only cells from subjects with DC demonstrated significant correlation. Telomere shortening in different cell types has been described previously in DC [4,6,7], however, the striking degree of correlation observed in the individual subjects examined in this study demonstrates the global defect in telomere maintenance in this disorder. Notably, relative telomere length was longer in fibro-blasts and buccal cells when compared to granulocytes across each specific IBMFS, perhaps reflecting the replicative histories of these cell types. Though significant intra-individual telomere length correlation between disparate tissue types has been observed in healthy subjects [8-13], few studies involving subjects with disease have evaluated telomere length in multiple tissues. By demonstrating such correlations within the DC population, Gadalla et al. provide evidence to support the use of relative telomere length in readily accessible cells such buccal or blood as a surrogate for inherent telomere maintenance capacity.

A higher proportion of single nucleotide variants within specific telomerase-associated genes, such as *TERT* and *TERC*, which encode the catalytic and RNA subunits of telomerase, respectively, have been described in cases with disease compared with a control population [14-19]. Interestingly, some variants have been described in familial cohorts with a predominant single phenotype, such as non-alcoholic liver disease or idiopathic pulmonary fibrosis, whereas the broader spectrum of the DC-associated phenotypes is not observed [16,20,21]. The impact of many of these variants on telomere maintenance has been inferred by measuring telomerase activity following transfection of the variant alleles into *TERT* or *TERC*-deficient cell lines, and by measuring telomere length, most often in leukocytes. Examining multiple tissue types in these cases of isolated phenotypes, similar to the approach of Gadalla, *et al*, may lend further support to the under-lying impact of these telomerase gene variants on constitutional telomere length maintenance.
